# A semi-field evaluation in Thailand of the use of human landing catches (HLC) versus human-baited double net trap (HDN) for assessing the impact of a volatile pyrethroid spatial repellent and pyrethroid-treated clothing on *Anopheles minimus* landing

**DOI:** 10.1186/s12936-023-04619-x

**Published:** 2023-07-03

**Authors:** Élodie A. Vajda, Manop Saeung, Amanda Ross, David J. McIver, Allison Tatarsky, Sarah J. Moore, Neil F. Lobo, Theeraphap Chareonviriyaphap

**Affiliations:** 1 Malaria Elimination Initiative, University of California, 550 16th street, San Francisco, CA 94158 USA; 2 Swiss Tropical and Public Health Institute (Swiss TPH), Kreuzstrasse 2, 4123 Allschwil, Switzerland; 3 University of Basel, Petersplatz 1, CH-2003 Basel, Switzerland; 4 Vector Control Product Testing Unit, Department of Environmental and Ecological Sciences, Ifakara Health Institute, P.O. Box 74, Bagamoyo, Tanzania; 5 Nelson Mandela African Institute of Science and Technology (NM-AIST), P.O. Box 447, Tengeru, Tanzania; 6 University of Notre Dame, Notre Dame, IN 46556 USA; 7 Kasetsart University, 50 Thanon Ngamwongwan, Lat Yao, Chatuchak, Bangkok, 10900 Thailand

**Keywords:** Human landing catches, Human-baited double net trap, Trap evaluation, Semi-field system, Bite prevention interventions, *Anopheles minimus*

## Abstract

**Background:**

The mosquito landing rate measured by human landing catches (HLC) is the conventional endpoint used to evaluate the impact of vector control interventions on human-vector exposure. Non-exposure based alternatives to the HLC are desirable to minimize the risk of accidental mosquito bites. One such alternative is the human-baited double net trap (HDN), but the estimated personal protection of interventions using the HDN has not been compared to the efficacy estimated using HLC. This semi-field study in Sai Yok District, Kanchanaburi Province, Thailand, evaluates the performance of the HLC and the HDN for estimating the effect on *Anopheles minimus* landing rates of two intervention types characterized by contrasting modes of action, a volatile pyrethroid spatial repellent (VSPR) and insecticide-treated clothing (ITC).

**Methods:**

Two experiments to evaluate the protective efficacy of (1) a VPSR and (2) ITC, were performed. A block randomized cross-over design over 32 nights was carried out with both the HLC or HDN. Eight replicates per combination of collection method and intervention or control arm were conducted. For each replicate, 100 *An. minimus* were released and were collected for 6 h. The odds ratio (OR) of the released *An. minimus* mosquitoes landing in the intervention compared to the control arm was estimated using logistic regression, including collection method, treatment, and experimental day as fixed effects.

**Results:**

For the VPSR, the protective efficacy was similar for the two methods: 99.3%, 95% CI (99.5–99.0) when measured by HLC, and 100% (100, Inf) when measured by HDN where no mosquitoes were caught (interaction test p = 0.99). For the ITC, the protective efficacy was 70% (60–77%) measured by HLC but there was no evidence of protection when measured by HDN [4% increase (15–27%)] (interaction test p < 0.001).

**Conclusions:**

Interactions between mosquitoes, bite prevention tools and the sampling method may impact the estimated intervention protective efficacy. Consequently, the sampling method must be considered when evaluating these interventions. The HDN is a valid alternative trapping method (relative to the HLC) for evaluating the impact of bite prevention methods that affect mosquito behaviour at a distance (e.g. VPSR), but not for interventions that operate through tarsal contact (e.g., ITC).

## Background

**Fig. 1:**
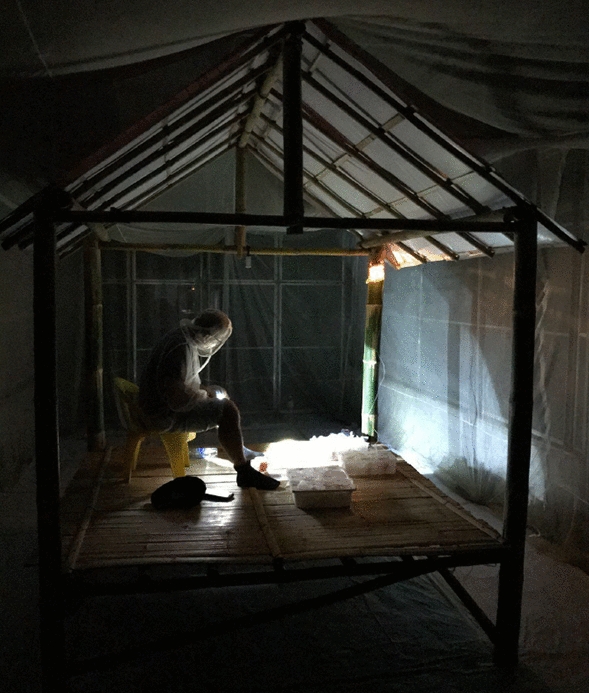
Human landing catch set-up in the temporary structure

The human-mosquito contact rate is an essential parameter for determining disease risk in a given area and reducing human-mosquito exposure is essential for reducing the risk of diseases such as malaria [1, 2]. Currently, mosquito landing is the accepted endpoint to measure the impact of mosquito bite prevention tools on human-vector exposure [3, 4]. It is typically measured by the human landing catch (HLC) method**,** the gold standard in both the field and the semi-field systems (SFS) [1, 3, 5]. To conduct HLCs, the collector manually or mechanically aspirates mosquitoes as they land on their exposed lower legs before the mosquito is able to bite (Fig. 1). However, HLCs are labour-intensive, expensive, and raise safety concerns about potential exposure of collectors to vector-borne diseases, such as dengue and malaria [5, 6]. While disease risk can be reduced by administering malaria prophylaxis to collectors [7], full protection cannot be assured in settings where drug resistance is an issue, and where mosquitoes carry other pathogens, such as arboviruses [8]. Consequently, some national malaria programmes (NMPs) do not allow for HLCs to be conducted, while other NMPs are currently working towards phasing out HLCs by replacing them with alternative adult mosquito collection methods [5].

In response to these challenges, a variety of possible alternative, ‘exposure-free’, collection methods been developed and evaluated against HLCs to examine mosquito landing rate equivalencies under semi-field and field conditions [9–12]. A commonly used alternative trap type is the CDC light trap [13], which is placed next to a person sleeping under a bed net. This method is easy to implement indoors, but may not accurately reflect human-vector exposure, whether used outdoors [14–16] or indoors [17]. Other collection methods that utilize humans as bait without requiring human-vector contact, include the human-baited double net trap (HDN) [18], the Ifakara tent trap [9], the Furvela tent trap [9], and the mosquito electrocuting trap [8], while the Suna trap utilizes a synthetic blend of chemicals that occur on human skin [19]. Amongst these traps, the HDN has shown promise in its potential to accurately measure the human landing rate [18, 20].

**Fig. 2:**
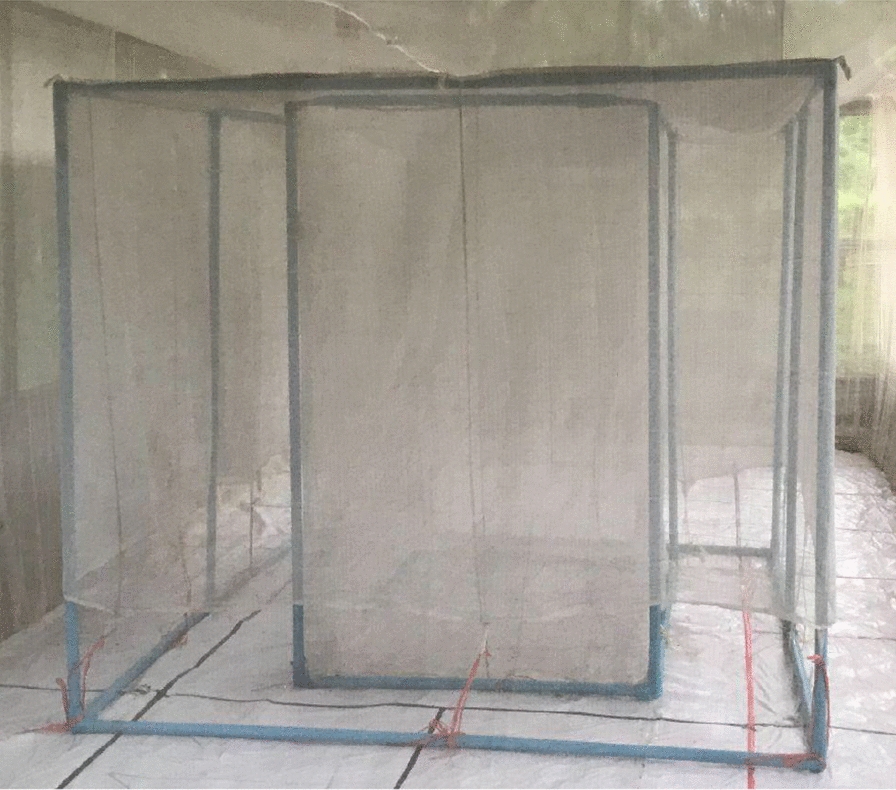
Human-baited double net trap structure

The HDN is a collection method that consists of a person sitting underneath two untreated nets, thereby preventing any human-mosquito contact (Fig. 2) [18]. In field evaluations in Lao PDR and Ethiopia, the HDN was tested against the HLC to compare human landing rates in the field for estimating human-vector exposure. Both studies found that the HDN collected similar numbers of *Anopheles* as the HLC. Specifically in the Lao evaluation, both the HLC and HDN capture rates were found to be comparable at both high and low mosquito densities in the absence of interventions [18, 20]. Also, a field study in Malaysia for evaluating the trapping densities of the HDN (and other trap types) compared to the HLC found that the HDN collected significantly less *Anopheles* than the HLC [21]. Further, in Thailand, a recent SFS evaluation of the performance of the HDN compared to the HLC for monitoring *Anopheles minimus* complex showed that the HDN captured significantly fewer mosquitoes than the HLC, indicating that the HDN might not be a suitable alternative to the HLC in this context [22]. Given this conflicting body of evidence on the suitability of the HDN as an alternative to the HLC, additional HDN evaluations are needed to help bring clarity around use of the HDN as a trap alternative to the HLC. Further, the HDN has not yet been tested in the presence of bite prevention interventions. To assess the efficacy of bite prevention tools, the HDN needs to be able to estimate the landing rates in both the control and the intervention arms. This can be a challenge because new bite prevention tools have varying modes of action which interfere with different mosquito behaviours [23] and trapping methods may have systematic biases [20, 24–26] that might affect the ability of the HDN to accurately reflect the HLC. This is because as opposed to the HLC, the HDN does not enable attracted mosquitoes to directly land on the collector, which may impact the measurement of protective efficacy of an intervention. Additionally, the HDN may affect the volatility of the volatile pyrethroid, concentrating the spatial repellent active ingredient within the netting enclosure around the collector, reducing the long-range activity of this intervention, thus interfering with the number of attracted mosquitoes [27].

There are two promising tools designed to protect the user from mosquito bites. First, spatial repellents (SRs), in particular, volatile pyrethroid spatial repellents (VPSRs) work by preventing human-vector contact primarily through non-contact irritancy (also referred to as non-contact excitorepellency or spatial repellency), landing inhibition, feeding inhibition, and sublethal incapacitation [28]. VPSRs are increasingly recognized as having important potential for public health use [29] and have been extensively evaluated in the SFS [27, 30–35] and in field experiments [36–38]. Second, insecticide-treated clothing (ITCs), treated with pyrethroids, primarily protect humans from mosquito bites through contact irritancy (also referred to as contact excitorepellency), some short-range non-contact excitorepellency, or feeding inhibition [28, 39]. ITCs have been extensively evaluated and show promise for their use against *Anopheles* biting amongst mobile populations and military/ranger personnel [29, 39–42].

Therefore, this study hypothesizes that the utility of the HDN may differ when the collector uses interventions that act primarily through contact irritancy versus spatial repellency in the vapour phase. In a semi-field system (SFS) in Sai Yok District, Kanchanaburi Province, Thailand, this study aims to assess whether the HDN can be used as a replacement collection method for the gold standard HLC in an outdoor setting to measure the protective efficacy against *An. minimus* landing of two types of mosquito bite prevention tools characterized by contrasting modes of actions: a) transfluthrin-based VPSR (non-contact irritancy), and b) etofenprox-treated ranger uniform (Eto R) (contact irritant). In Southeast Asia, *An. minimus *sensu lato is an important malaria vector [43–45] and is, therefore, a highly relevant *Anopheles* species to include in this evaluation.

## Methods

### Study site

**Fig. 3:**
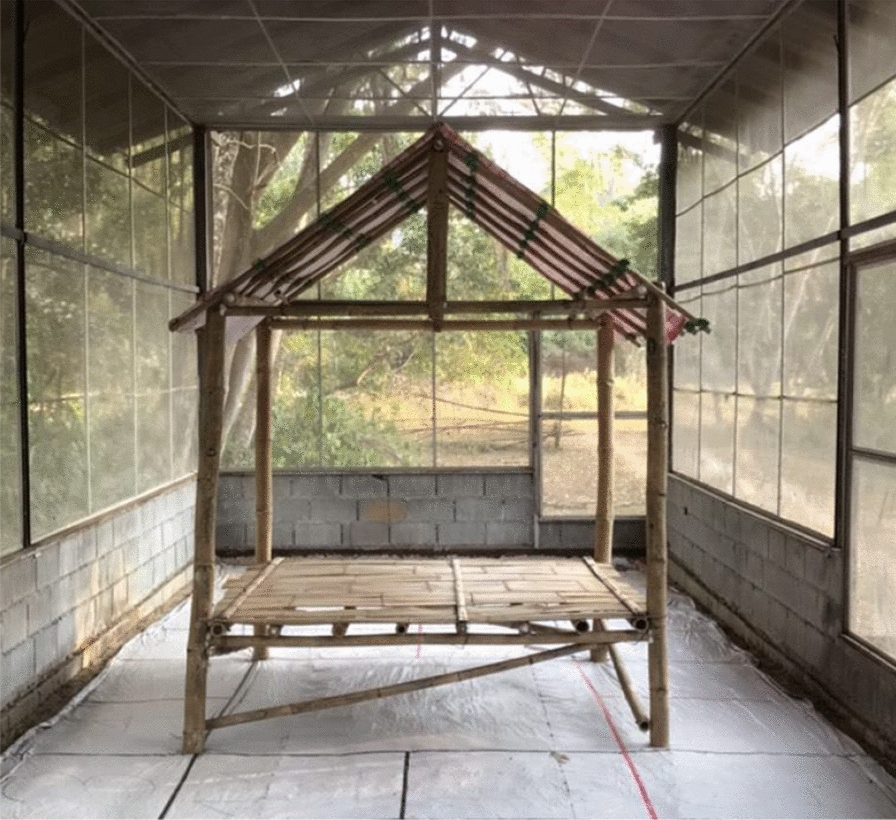
Bamboo temporary, open structures at Kasetsart University (KU)

This evaluation was conducted in Kasetsart University’s SFS in Pu Teuy Village, Sai Yok District, Kanchanaburi Province, Thailand. Experimental chambers (9 × 4 × 3 m) of the SFS structure were positioned at least 20 m apart to minimize any spillover effect where the chamber containing the intervention impacts the behaviour of mosquitoes in the chamber containing the control. Temporary open structures (2 × 2 × 2 m), designed to mimic typical temporary shelters used by people in forested settings across southeast Asia, were constructed inside each experimental chamber (Fig. 3). Each open structure consisted of four bamboo poles (2 m) with a tarpaulin placed/attached to the top with an overhang of 30 cm.

### Mosquito bite prevention interventions

Two interventions were included in this evaluation. The first is a transfluthrin-based VPSR that was hung from the open structure’s eaves. For this evaluation, two units of this product were hung from two opposite sides of the open structure, per manufacturer’s instructions. Note, due to limited space in the chambers, the HDN structure was not placed over the open structure and was tested without the presence of the temporary shelter. Thus, the VPSR devices were hung from the HDN’s opposing poles supporting the outer net. As the product manufacturer recommends replacing the product once every 30 days, the product was replaced after two experimental blocks (of eight days) to minimize possible waning efficacy. The second intervention was etofenprox treated ranger uniforms (Eto R). The ranger uniforms were hand-treated with etofenprox at Kasetsart University according to the manufacturer’s instructions: the bottle was held 15–20 cm away from garment to allow spraying on fabric for a treatment level of 2.0 g/m^2^. Using slow, sweeping motions, the garments were evenly coated for approximately 30 s on each side. Garments were air dried for two hours by being hung outside. Each collector had their own treated ranger uniform, and these were not washed throughout this study, and freshly treated ranger uniforms were used for each experimental block to minimize possible waning efficacy. Collectors in the VPSR intervention and control arms wore short sleeves and short trousers, along with a net jacket, leaving the area between the knees and ankles exposed. Collectors in the Eto R intervention and control arms wore treated (intervention) or untreated (control) ranger uniforms (long sleeves and long trousers), along with a net jacket, and for HLCs, mosquitoes were collected from the area between the knees and ankles as they landed on the clothing. In this way, the additional effect on mosquito landing of the insecticide could be measured.

### Experimental design

**Fig. 4:**
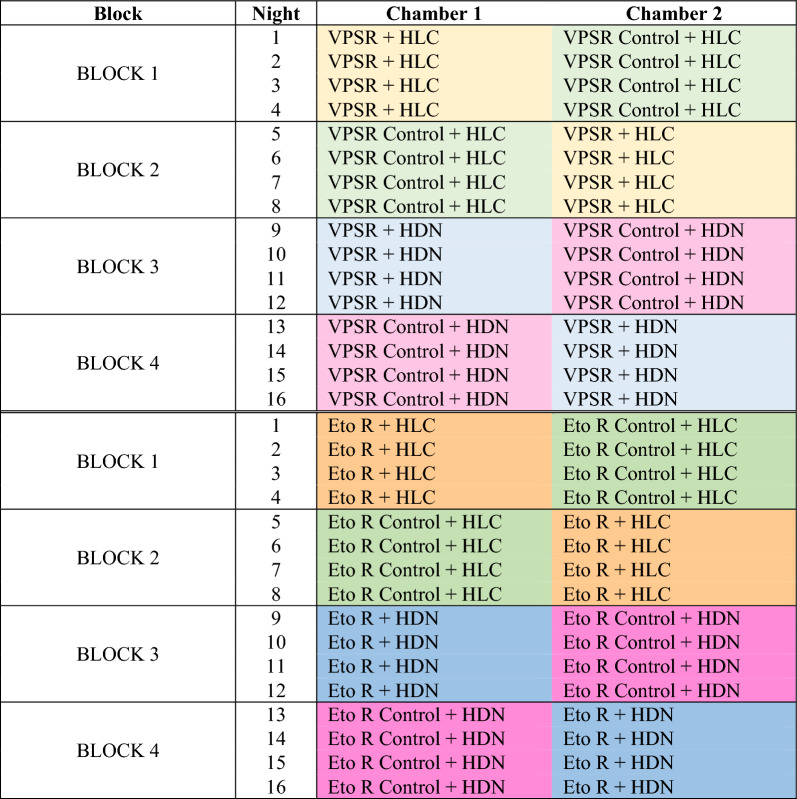
Experimental design of the trap evaluation. **a** Number of mosquitoes collected per arm, per night via HDN and HLC when using the VPSR (eight replicates (one replicate = a 6 h collection night) per VPSR and control arm). **b** Number of mosquitoes collected per arm, per night when wearing Eto R via HDN and HLC (eight replicates per Eto R and control arm)

The evaluation was conducted over 32 nights using a block randomized cross-over design with 16 nights of collection for each of the two interventions (Fig. 4). The study consisted of two experiments. The first experiment evaluated the protective efficacy of the VPSR measured by HLC versus HDN, and the second experiment evaluated the protective efficacy of etofenprox treated clothing (Eto R) measured by HLC versus HDN. There were four arms for each of the two interventions: (1) HLC in the presence of intervention (treatment), (2) HLC in the absence of intervention (control), (3) HDN in the presence of the intervention (treatment), (4) HDN in the absence of the intervention (control). Treatments and controls were randomly allocated to either chamber of the SFS and remained in that compartment for a four-night experimental block. Four collectors conducted the experiments and switched between chambers on a nightly basis to control for any bias caused by individual attractiveness to mosquitoes that may affect repellent efficacy. After 16 test nights were completed, each collector had evaluated each of the study arms four times in each of the two chambers (Fig. 2). At the end of each experimental block, chambers were ‘washed out’ by allowing chambers to sit for two days with no activity. Successful wash out of active ingredients was verified by running a cone bioassay [46] on the walls of the chamber and of the open structure roofs using a pyrethroid susceptible mosquito strain. If no vector knock down was observed, then the compartment was designated as clean. After this two-night wash out period, the treatment allocation was switched (crossed over). For each intervention, during the first two blocks, HLCs were conducted and in the second two blocks the HDN was used.

### Mosquitoes and mosquito collection

KU’s *An. minimus* laboratory (L) strain is originally from Rong Klang district, Prae province, Thailand, and has been reared at KU’s insectary for the purpose of SFS studies since 1993 [22]. For this study, female *An. minimus,* nulliparous and aged five to eight days old, were sugar starved for eight hours prior to release in the SFS to ensure avidity to host seeking. Susceptibility to pyrethroids (permethrin, deltamethrin, transfluthrin, etofenprox) was confirmed prior to this study with 1 × discriminating dose in the World Health Organization insecticide susceptibility bioassay [47]. For transfluthrin, papers were treated according to Sukkanon et al*.* [48]. Each collection night consisted of one six-hour replicate (20h00 to 02h00). Head torches were used by the collector to aid the collection of mosquitoes. Mosquitoes were transferred from the insectary to a holding chamber 30 min before the experiment was initiated to acclimate. The holding chamber was kept separate from the experimental chamber where the product being evaluated was present. For each replicate, a single release of 100 mosquitoes was done at 20h00 in each chamber.

HLCs were conducted for 45 min during each hour of the six-hour replicate, allowing for a 15-min break per collection hour. To conduct the HLCs, a collector sat on a chair in the centre of the temporary structure and collected mosquitoes with a mouth aspirator as mosquitoes landed on the lower leg between the knee and the ankle, before the mosquitoes were able to bite (Fig. 1).

The HDN consists of a person sitting underneath two untreated, white nets: the first net is sealed against the ground and protects the human from direct mosquito exposure, and the second, larger net is placed directly over the inner net (Fig. 4). The larger, outer net, is raised slightly above the ground (30 cm) to allow mosquitoes attracted to the human ‘bait’ to enter the space between the outer and inner net. Trapped mosquitoes are collected from this space [18]. The HDN structure measured: inner net: 80 cm (width) × 150 cm (length) × 150 cm (height); outer net: 180 cm (width) × 250 cm (length) × 120 cm (height)) (Fig. 4). The collector sat inside the HDN for 45 min of each hour, and then conducted aspirations of mosquitoes resting between the inner and outer nets of the HDN structure for the final 15 min of the hour with collection cups labelled for 1-h intervals. HLC and HDN collection cups were replaced every hour and cups containing collected mosquitoes were placed into a plastic box to avoid further exposure to insecticides before transfer to the insectary at the end of the 6-h experiment.

### Data analysis

The OR of the released *An. minimus* mosquitoes landing in the intervention compared to the control arm was estimated using logistic regression. Fixed effects included collection method (HLC or HDN), treatment (intervention or control), and experimental day. The model initially included a random effect for the batch of mosquitoes (defining a batch as all mosquitoes released together in one chamber on one night), but due to the large number of zero landings, the parameters could not be estimated and the random effect was removed. To directly assess the difference in the estimated effect by trap type, an interaction term between intervention and collection method was included as a fixed effect. Due to the limited number of replicates (eight per arm), covariates for chamber and volunteer were not included but this is not expected to have a substantial impact since the study had a fully balanced design (each treatment occurred an equal number of times in each sequence, and each collector received each treatment an equal number of times). All estimates are presented with 95% confidence intervals (CI). The analysis was conducted in R [49] using the tidyverse packages ‘tidyr’, ‘dplyr’, ‘ggplot2’, and ‘lme4’. Protective efficacy was estimated as (1-OR)*100.

## Results

**Fig. 5:**
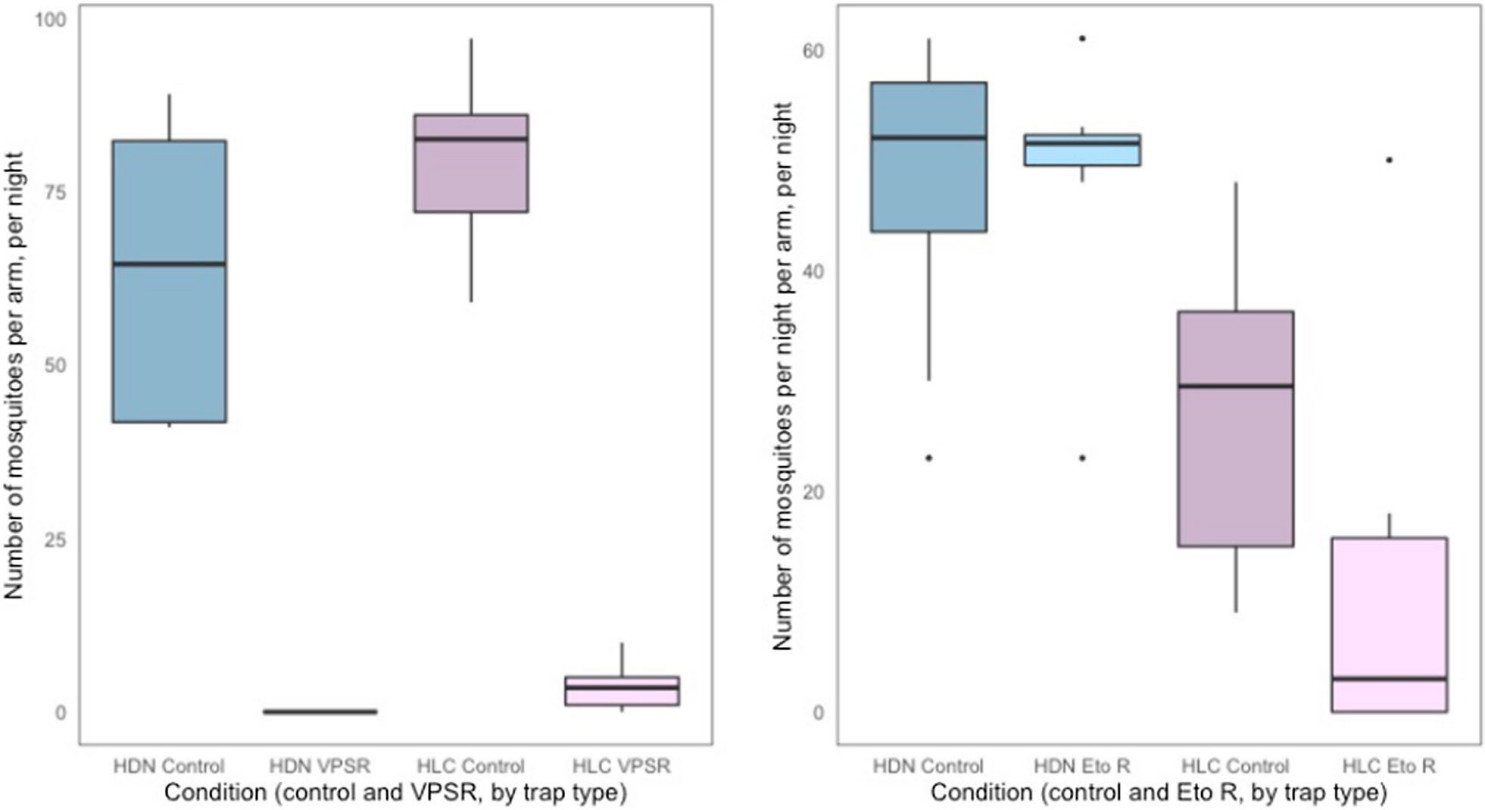
Box plots of the number of mosquitoes collected per arm (control, intervention), per night (100 *An. minimus* released per replicate) when collecting with the human-baited double net trap (HDN) and the human landing catch (HLC)

When using the VPSR, the HLC and the HDN yielded similar mosquito captures; Median (Mdn) number of mosquitoes captured per night was of 3.5 (IQR = 1–5) via HLC, and of 0 (IQR = 0–0) via HDN (Fig. 5a). In contrast, when using the Eto R, the HLC and the HDN did not yield similar mosquito captures; Mdn number of mosquitoes captured per night was of 3.0 (IQR = 0–15.75) via HLC, and of 51.5 (IQR = 49.5–52.25) via HDN (Fig. 5b).

In the control arms, there was some variability in the mosquito recapture. For the VPSR control arms (Table 1, Fig. 5a) HLC control numbers were higher than those for HDN control (OR 2.18, 95% CI (1.74–2.72), p < 0.001). Whereas, for the Eto R control arms (Table 1, Fig. 5b), HLC control numbers were lower than those for HDN control (OR 0.42, 95% CI (0.34–0.51), p < 0.0001).

**Table 1:** Estimated impact of trap type, intervention status, and the interaction between trap type and intervention status on An. minimus catches, for Eto R and VPSR.

	Eto R				VPSR			
	Factor	OR (95% CI)	p	% protective efficacy	Factor	OR (95% CI)	p	% Protective efficacy
Mosquito captures in control HLC compared to control HDN	HDN	1			HDN	1		
	HLC	0.42 (0.34–0.51)	<0.0001		HLC	2.18 (1.74 – 2.72)	< 0.001	
Effect of intervention on landing when using HLC	Control	1			Control	1		
	ITC	0.30 (0.23, 0.40)	< 0.001	70% (60–77) **decrease** in odds of landing	VPSR	0.007 (0.005, 0.01)	< 0.001	99.3% (99.0–99.5) **decrease** in odds of landing
Effect of intervention on landing when using HDN	Control	1			Control	1		
	ITC	1.04 (0.85, 1.27)	0.69	4% (15% decrease to 27% **increase** in odds of landing)	VPSR	8.72e-14 (0, Inf) 0 mosquitoes were collected via HDN across all 8 replicates when using VPSR	0.99	100% (100 -Inf) decrease in odds of landing
OR for intervention measured by HDN compared to OR for intervention measured by HLC (ratio of ORs)	ITC	3.17 (2.27, 4.43)	< 0.001		VPSR	3.21 (0, Inf)	0.99	

The protective efficacy of the bite prevention interventions was estimated by HLC and HDN. The protective efficacy for the VPSR was 99.3% (99.5–99) when measured by HLC (OR 0.007 (0.005, 0.01), p < 0.001), and 100% measured by HDN as no mosquitoes were recaptured (Table 1, Fig. 5a. The protective efficacy of Eto R was 70% (60–77) when measured by HLC (OR 0.30 (0.23, 0.40), p < 0.001), but there was no evidence of an effect when measured by HDN (OR 1.04 (0.85, 1.27), p = 0.69) (Table 1, Fig. 5b).

The ORs estimated by HLC and HDN for the VPSR were both very small, and lead to the same conclusion of the impact of the VPSR against mosquito landing, which is that the VPSR tested is highly protective against mosquito landing. Conversely, the OR estimated by HDN for Eto R was an estimated 3.17 times (2.27–4.43) p < 0.001 that of the OR estimated by the HLC, and would lead to opposing conclusions on the protective efficacy of Eto R against mosquito landing. Table 1 Estimated impact of trap type, intervention status, and the interaction between trap type and intervention status on *An. minimus* catches, for Eto R and VPSR.

## Discussion

This study evaluated the performance of two different mosquito trapping methods, the HLC and the HDN, for estimating the effect on mosquito landing rates of two intervention types characterized by contrasting modes of action. The study results indicated that the primary mode of action of each intervention had a substantial impact on how the HDN performed in comparison to the HLC to evaluate the protective efficacies of the VPSR and the etofenprox-treated clothing. The VPSR works primarily through non-contact irritancy; mosquitoes move away from the VPSR or become incapacitated at a distance from the VPSR, without coming into physical contact with the treated substrate. In contrast, Eto R primarily works through contact irritancy, requiring tarsal contact with the treated surface for mosquitoes to be affected by the active ingredient.

When evaluating the VPSR intervention, the direction of the estimated effects of the intervention on mosquito landing is similar when measured by HLC and by HDN. However, the HDN intervention arm collected zero mosquitoes, which meant that a precise effect could not be estimated. This observation suggests that a larger number of replicates than the eight included in this study would be necessary when using the HDN to estimate the impact of a VPSR. However, given the magnitude of the observed efficacy, it can be concluded that the HDN remains a suitable collection method for evaluating the protective efficacy of VPSRs.

When evaluating the Eto R intervention, there was no evidence of an effect of Eto R on mosquito landing obtained via HDN. In contrast, when using the HLC, the estimated effect of Eto R demonstrated a strong reduction in mosquito landing. The HDN is not a suitable collection method for ITCs likely because this intervention functions primarily through contact irritancy, which requires mosquitoes to physically land on the treated material worn by the collector to then be repelled by the active ingredient [50, 51]. As the HDN method does not allow the mosquito to come into direct contact with the collector, the mosquito is not repelled by the intervention, and is captured by the HDN. It can also be reasonably expected that this may also be the case for topical repellents. Synthetic topical repellents such as DEET and picaridin, are applied to the skin to provide a surface barrier against mosquitoes. Similarly to ITCs, topical repellents also provide personal protection against mosquito bites through a range of behavioural actions at short-range actions, such as olfactory attractor inhibition (as is the case with DEET) [52, 53], mosquito diversion through non-contact excitorepellency, and/or through contact irritancy of mosquitoes that have made tarsal contact with the treated surface [54, 55]. As the HDN blocks short-range activity, the HDN is also likely an unsuitable trapping method to evaluate the protective efficacy of topical repellents.

To date, mosquito landing is the accepted endpoint to evaluate the impact of bite prevention tools on human-vector exposure [3], measured by the gold standard HLC trapping method. However, a gap exists in the collective repertoire of proven field collection methods other than HLCs to evaluate the impact of interventions that act primarily through non-contact or contact irritancy (spatial repellency) on mosquito landing (i.e., human-vector exposure) [12]. There are several SFS and field intervention evaluation studies that have evaluated the impact of VPSRs on mosquito landing as a proxy for biting using trapping methods other than the HLC, such as the CDC LT [56–59]. However, there have been relatively few SFS intervention evaluations that have directly compared the estimated protective efficacy against mosquito landing obtained from HLCs versus alternative trapping methods. Recently, Swai et al. (Pers. Comm.) compared the protective efficacies against mosquito landing obtained when using HLCs versus CDC LTs to evaluate the impact of transfluthrin VPSRs on estimates of exposure to mosquitoes, and found that CDC LT is not a proxy for HLC for the evaluation of VPSRs. Other studies of transfluthrin-based VPSRs using the mosquito electrocuting trap and the BG Sentinel trap with *Aedes aegypti* showed that they could only be used to estimate the protective efficacy if used independent of competing sources of kairomones (semiochemicals associated with mosquito-host interactions) [60].

While there is the opportunity to conduct HLCs in a safe manner using disease-free mosquito populations in the SFS, there remains a pressing need to validate alternative methods to HLCs for evaluating ITCs and VPSRs, particularly outside the SFS, as many countries do not permit HLCs in the field. A limitation of this study is that this evaluation was conducted with a single species (*An. minimus*) and within the highly controlled environment of an SFS. In the wild, this species complex displays heterogenous levels of anthrophily and endophagy across the region [44, 45]. As different mosquito species are characterized by specific behaviour profiles (e.g., varying levels of anthropophagy) and pyrethroid resistance mechanisms which uniquely affect intervention functionality [53, 61–65], alternative trap types to the HLC should always be evaluated against a given site’s local vectors before it is employed as a proxy for the HLC in the field. Thus, findings from this trap evaluation could be further corroborated by conducting additional trap evaluation studies such as this one in the SFS with different mosquito species and resistance mechanisms, and in various field settings. In addition, the construct of the HDN should be standardized across all studies, such that the exact same type of HDN is tested in other evaluations.

Finally, this study could be further strengthened by conducting evaluations throughout the life of the VPSR intervention so that more reliable estimates of comparative efficacy can be made as the product wanes in efficacy and, presumably, more mosquitoes are captured. As VPSR efficacy wanes and more mosquitoes are attracted to the host, it is possible the estimated protective efficacy via HDN indicates less protection against mosquito landing than the estimated protective efficacy via HLC. This is because the HDN’s inner netting enclosure might further concentrate the VPSR’S active ingredient within the netting enclosure around the collector, interfering with the long-range activity of this intervention, thus further decreasing the number of attracted mosquitoes compared to the HLC [27].

## Conclusion

This study indicates that HDNs may be a suitable replacement for HLC for evaluating VPSRs in the field. However, for evaluating ITCs and other similarly acting interventions that function through short range modes of action and tarsal contact (such as, topical repellents), there are no known, validated alternative trapping methods to the HLCs. Therefore, other field collection methods should be evaluated to identify alternatives that would enable field evaluations of mosquito bite prevention products functioning through contact irritancy on mosquito landing in areas where HLCs are not possible.

## Data Availability

Data supporting the analysis, outcomes, and conclusions of this article are available upon request to the corresponding author.

## Abbreviations

CDC LTs: Center for Disease Control Light Traps
Eto R: Etofenprox-treated ranger uniform
HDN: Human-baited double net trap
HLC: Human landing catches
ITC: Insecticide-treated clothing
Mdn: Median
NMPs: National malaria programmes
SFS: Semi-field system
VPSR: Volatile pyrethroid spatial repellent
